# A qualitative and quantitative exploration of determinants and intervention methods for increasing condom use among internal migrants in China: results of a three-round Delphi study

**DOI:** 10.1186/1471-2334-14-S2-P33

**Published:** 2014-05-23

**Authors:** Xiaona Liu, Vicki Erasmus, Xinying Sun, Rui Cai, Yuhui Shi, Jan Hendrik Richardus

**Affiliations:** 1 Erasmus MC, University Medical Center Rotterdam, Rotterdam, The Netherlands

## Introduction

Internal migrants have been repeatedly characterized as “the tipping point” for HIV epidemic in China. This study is a step towards the development of a successful behavioral HIV prevention intervention among Chinese internal migrants. It aims to explore important and changeable determinants of condom use, and inspect effective and feasible methods to increase condom use for the target population.

## Materials and methods

We conducted a three-round web-based Delphi study among a panel of 62 experts between October 2012 and March 2013. In the first round, the panelists were asked to qualitatively identify determinants of condom use, and past and/or ongoing relevant intervention methods they considered successful. In the subsequent rounds, they were asked to rate importance and changeability for each identified determinant, and to rate effectiveness and feasibility for each identified intervention method, on a 5-point Likert scale. The median and Inter Quartile Deviation (IQD) were calculated for describing each item. Items with an IQD ≤ 1 were judged as reaching consensus among the panelists.

## Results

Overall, 21% of our initial sample completed all three rounds of the study (13). The panelists identified 19 possible determinants of condom use, and they considered 16 intervention methods successful. The quantitative results were obtained from the panelists with a variety of expertise across the globe, but reached strong consensus over almost all questions (98%). They agreed that attitude of towards using condoms were important (median=5) and changeable (median=4) determinants of condom use, while applying behavioral theory, increasing education and condom access, performing worksite health promotion, detecting risk factors, and working closely with relevant organizations and the government were effective (median=5) and feasible (median=4) methods to increase condom use among internal migrants in China.

## Conclusions

The development of a successful behavioral HIV prevention intervention targeting Chinese internal migrants should build messages upon a behavioral theory integrating multiple determinants (e.g., cognitive factors, skills, social influence), with the priority of shaping positive attitude towards condom use among the target population, and should take multiple identified intervention methods (e.g., education, condom access, worksite health promotion) into consideration.

